# Cardiovascular Disease Mortality Patterns Among People With Cancer in New South Wales, Australia: A Population‐Wide Data Linkage Study

**DOI:** 10.1002/cam4.71568

**Published:** 2026-03-06

**Authors:** Md Mijanur Rahman, Karen Canfell, Katy Bell, Grace Joshy, Michael David, Anne Cust, David Goldsbury, Bogda Koczwara, Emily Banks, Xue Qin Yu

**Affiliations:** ^1^ Collaboration for Cancer Outcomes Research and Evaluation (CCORE), School of Clinical Medicine University of New South Wales (UNSW) Sydney Australia; ^2^ The Daffodil Centre The University of Sydney, A Joint Venture With Cancer Council NSW Sydney Australia; ^3^ Sydney School of Public Health University of Sydney Sydney Australia; ^4^ National Centre for Epidemiology and Population Health Australian National University Canberra Australia; ^5^ School of Medicine and Dentistry Griffith University Gold Coast Australia; ^6^ Flinders Health and Medical Research Institute, College of Medicine and Public Health Flinders University Adelaide Australia; ^7^ Australian Research Centre for Cancer Survivorship Faculty of Medicine and Health, UNSW Sydney Australia

**Keywords:** absolute excess risk, cardiovascular disease, people with cancer, standardised mortality ratio

## Abstract

**Objective:**

To investigate cardiovascular disease (CVD) mortality patterns among people with cancer in New South Wales (NSW) compared to the NSW general population and trends over time.

**Design:**

Population‐wide retrospective data‐linkage study.

**Participants and Setting:**

873,344 people aged ≥ 40 diagnosed with primary invasive cancer and registered in the NSW Cancer Registry between 1985 and 2019.

**Main Outcomes:**

CVD as the underlying cause of death. Absolute mortality rate (AMR, per 10,000 person‐years) and standardised mortality ratios (SMR) compared to the NSW general population.

**Results:**

Of 514,865 people with cancer who died between 1985 and 2020, 71% (*n* = 303,770) died from cancer and while 14% (*n* = 73,733) from CVD. AMR of CVD increased with time since cancer diagnosis (≤ 2 years: AMR = 124 vs. > 10 years: AMR = 152) and declined from 188 in 1985–1995 to 68 in 2010–2020 following cancer diagnosis in 1985–1989 and 2010–2014, respectively. Approximately 45% of CVD deaths were due to ischaemic heart disease, with 49% in males versus 40% in females. Elevated SMRs of CVD were found for ≤ 2 years after cancer diagnosis (SMR = 1.17, 95% CI: 1.15, 1.19), those aged 40–59 (SMR = 1.13, 95% CI: 1.07, 1.19) and those diagnosed with distant metastases (SMR = 1.31, 95% CI: 1.27, 1.35) or lung cancer (SMR = 1.42, 95% CI: 1.37, 1.47). Lower CVD SMRs were observed for > 2 years after cancer diagnosis (e.g., for 2–5 years: SMR = 0.84, 95% CI: 0.83, 0.86), among people diagnosed with cancers with higher survival rates (e.g., Melanoma: SMR = 0.79, 95% CI: 0.78, 0.81) and in the most recent period 2010–2020 (SMR = 0.85, 95% CI: 0.83, 0.87).

**Conclusion:**

Higher than expected CVD mortality compared to the general population was observed within the first few years after cancer diagnosis, especially among those diagnosed with lung cancer and distant metastases, which might reflect shared risk factors and cardiotoxic treatment. Our findings underscore the need for CVD risk assessment, prevention, and care among people with cancer.

## 1 Introduction

Cancer and cardiovascular disease (CVD) are the leading causes of death in Australia [1] and other high‐income countries [2, 3]. Despite improvements in cancer survival rates over the past few decades, there is strong evidence that a new cancer diagnosis and its treatment‐related side effects can exacerbate preexisting conditions and introduce new conditions [4, 5, 6]. A substantial proportion of people with cancer are likely to die from other conditions, particularly CVD [3]. High underlying rates of CVD mortality in the general population are increased further in people with a cancer diagnosis. One reason for this is that many of the common cancer types and CVD have shared risk factors; for example, smoking, obesity, and socioeconomic disadvantage, and those with such risk factors are at increased risk for both conditions [7, 8]. Additionally, the cardiotoxicity of many anti‐cancer treatments, such as chest radiation and chemotherapy, may also increase CVD risk among people with cancer [5, 9, 10, 11], though there have been considerable efforts to reduce such cardiotoxicity with recent advancements in radiotherapy schedules and cardioprotective strategies [12]. With a growing population of cancer survivors [13], understanding the complex dynamics between these two diseases is a public health priority.

Previous studies report an elevated risk of CVD in cancer survivors compared to the general population, but variations exist across cancer type, age at diagnosis, and time since diagnosis [5, 14, 15, 16]. Variation may also exist over time (calendar year), given improvements in cardiovascular prevention and management in the general population, as well as the advancement in the management of cardiotoxicity from anti‐cancer treatments. A previous Australian study reported that 27% of all deaths in cancer survivors who survived five years after initial diagnosis were due to CVD, which is equivalent to 56% of all non‐cancer deaths [17]. Another study reported that the cumulative incidence of CVD deaths increased with age and exceeded other competing events (including primary cancer) in people with cancer aged 65 and over [18]. However, the follow‐up time of this study was relatively short and unable to examine CVD mortality trends for long‐term cancer survivors.

Despite the evidence for an increased CVD mortality risk in people with cancer compared to the general population, only a few population‐based studies have exclusively focused on CVD in people with cancer [5, 15, 18, 19]. There is a lack of consistency in the findings due to the heterogeneity in the study populations, follow‐up times, small size, and imprecise/nonsignificant estimates. Additionally, there has been a noticeable reduction in CVD mortality in the general population over the last few decades [20]. However, no study has yet examined the trend in CVD mortality among people with cancer in Australia. We aimed to quantify CVD mortality in people with cancer in New South Wales (NSW) compared to the NSW general population and assess the temporal trends in CVD mortality by major cancer types, time since cancer diagnosis, and calendar year period.

## 2 Methods

### 2.1 Data Source and Study Population

This study utilised data from the Cancer Institute NSW's Enduring Cancer Data Linkage (CanDLe), a multiple data linkage initiative for all people with cancer registered in the NSW Cancer Registry (NSWCR), which represents approximately one‐third of cancer diagnoses in Australia [21, 22]. The study population included 873,344 people diagnosed with primary invasive cancer at age ≥ 40 between 1985 and 2019. Information on mortality until 2020 was from the Australian Coordinating Registry's linked Cause of Death Unit Record File (COD URF), allowing a minimum of one year of follow‐up since cancer diagnosis. We excluded people with cancers that were diagnosed by autopsy or death certificate (*n* = 6951), people alive less than a day after the cancer diagnosis (*n* = 26,884), and people with an in situ cancer diagnosis only (*n* = 1851). We restricted our analysis to those aged 40 years and over as the incidence of CVD death is very low below this age.

We obtained ethics approval for this study from the NSW Population and Health Services Research Ethics Committee (Ref: 2023UMB0501). We also obtained data on CVD mortality rates in the NSW general population by age, sex and calendar year between 1985 and 2020 from the Australian Bureau of Statistics with a special request [23].

### 2.2 Incident Cancer Cases

People with an invasive primary cancer diagnosis (excluding keratinocyte skin cancers) between 1985 and 2019 were identified using the International Classification of Disease version 10 (ICD‐10) codes from the NSWCR dataset. The current study focussed on any malignancy (C00‐C96) and five major cancer types: prostate (C61), female breast (C50), colorectal (C18‐20), lung (C34) cancers and melanoma of the skin (C43), which together represent over 60% of all cancer diagnoses.

### 2.3 Underlying Causes of Death

The underlying cause of death was identified from the COD URF using the ICD‐9 codes for the period 1985 to 1996 and the ICD‐10 codes for the period 1997 to 2020. Causes of death were broadly classified into three groups: cancer death (ICD‐9: 140–208 and ICD‐10: C00‐C96), CVD death (ICD‐9: 390–459 and ICD‐10: I01‐I99) and other causes of death. CVD death was further categorised into four broad groups: (1) Ischaemic heart disease (IHD), (2) hypertensive, pulmonary and other heart disease, (3) cerebrovascular disease and (4) all other CVD deaths. Detailed ICD‐9 and ICD‐10 codes for different types of CVD deaths are provided in Table S1.

### 2.4 Statistical Analysis

Study participants were followed from the date of diagnosis of primary cancer to the end of the study (31 December 2020) or the date of death, whichever was the earliest. The population at risk was constructed for each calendar year, using age as the underlying timescale. Person‐years at risk were calculated from the date of cancer diagnosis to either the end of the study or death, with the time‐splitting done by running the well‐validated SAS macro‐Lexis.

Descriptive statistics were used to summarise broad categories of causes of death (deaths from cancer, CVD and other causes) and follow‐up time in the study population stratified by age at diagnosis for any malignancy and five major cancers. The absolute mortality rate (AMR) of CVD per 10,000 person‐years was calculated by time since cancer diagnosis. The proportion of deaths over time since diagnosis of cancer by causes of death was calculated for all cancers combined and five major cancers and presented by stacked bar charts to explore the changes during cancer survivorship. The distribution of cause‐specific CVD deaths was further examined by sex, age at death, and cancer type.

Age‐ and sex‐specific CVD mortality rates of the NSW general population in each calendar year were merged with the cancer cohort to compare the CVD mortality patterns. Standardised mortality ratio (SMR) and 95% confidence interval (CI) were calculated using the standard cohort technique to compare CVD mortality between people with cancer and the NSW general population [24, 25], stratified by time since cancer diagnosis and by cancer type. SMR is the ratio of the observed number of CVD deaths in the study population (during the time at risk) divided by the expected number of deaths in the study population based on the rates in the reference population (NSW general population). The expected number of deaths was calculated considering the number of person‐years at risk among the cancer cohort for each stratum of sex‐specific 5‐year age groups, one‐year calendar period, and the corresponding population mortality rate.

To examine the temporal trend in CVD deaths over the study period (1985–2020), we compared the AMR (per 10,000 person‐years) and proportion of CVD deaths for three sub‐cohorts of people who were first diagnosed with cancer in three different periods, including 1985–1989, 2000–2004, and 2010–2014. These three cohorts were followed up equally (6–11 years) until 1995, 2010 and 2020, respectively. SMRs and 95% CIs of CVD mortality stratified by sex, age at cancer diagnosis, the spread of cancer at diagnosis, calendar year and major cancers were calculated separately for each cohort. The analyses were performed in SAS 9.4 (SAS Institute Inc., Cary, NC).

## 3 Results

Of 873,344 people with cancer diagnosed between 1985 and 2019, 514,865 (59%) died by 31 December 2020, with a median follow‐up time of 8.1 years for those diagnosed with cancer at age 40–59, 4.5 years for age 60–79, and 1.7 years for age 80 years or over (Table 1). Overall, 71% of deaths were from cancer, 14% from CVD, and 15% from other underlying causes. The proportion of deaths due to CVD increased with age at diagnosis: 6% (*n* = 5547) of those aged 40–59 at the time of their cancer diagnosis to 20% (*n* = 23,266) of those aged 80 or over. The proportion of deaths due to cancer showed the opposite pattern, accounting for 84% and 62% of deaths, respectively. IHD accounted for the largest proportion of all CVD deaths, with a greater proportion among males (49% vs. 40% in females), younger patients (47% in age 40–59 vs. 43% for age > 80), and males with lung cancer (54%) (Table S2). The proportion of CVD deaths due to cerebrovascular disease was higher among females (15%) than males (11%).

**TABLE 1:** Characteristics of people diagnosed with primary invasive cancer between 1985 and 2019 in New South Wales, Australia (*n* = 873,344) with mortality follow‐up to 2020 by cause of death (*n* = 514,865).

Cancer type (ICD‐10 codes)	Age at diagnosis	Cancer cases^a^	Median follow‐up (years)	Number of deaths^b^	% of deaths by underlying cause		
					Cancer	CVD	Other
Any malignancy (C00‐C96)	40–59	248,783	8.1	92,451	84%	6%	10%
	60–79	488,856	4.5	306,086	70%	15%	15%
	≥ 80	135,705	1.7	116,328	62%	20%	18%
Prostate (C61)	40–59	24,049	9.8	3923	73%	12%	15%
	60–79	101,510	7.9	46,035	53%	24%	23%
	≥ 80	19,246	3.4	15,794	46%	30%	24%
Breast (C50)	40–59	54,874	10.9	13,273	84%	6%	10%
	60–79	52,310	8.2	22,993	53%	24%	23%
	≥ 80	11,840	4.0	9088	41%	33%	26%
Colorectal (C18‐C20)	40–59	26,206	10.5	11,687	85%	6%	9%
	60–79	67,355	7.8	43,431	65%	18%	17%
	≥ 80	22,704	4.2	18,802	58%	23%	19%
Lung (C34)	40–59	15,711	1.1	13,026	94%	2%	4%
	60–79	54,007	0.8	48,041	89%	5%	6%
	≥ 80	13,527	0.4	12,752	88%	6%	7%
Melanoma (C43)	40–59	32,179	13.1	6000	71%	13%	16%
	60–79	39,995	8.2	18,695	48%	27%	25%
	≥ 80	11,017	4.1	8113	34%	36%	30%
Other cancers	40–59	96,227	9.0	44,542	84%	5%	11%
	60–79	177,052	5.2	126,891	76%	11%	13%
	≥ 80	60,485	2.4	51,779	70%	15%	15%

Abbreviations: CVD, cardiovascular disease; ICD‐10, International Classification of Diseases, 10th Revision.

^a^ Diagnosed with cancer between 1985 and 2019.

^b^ Died between 1985 and 2020.

### 3.1 Absolute Mortality Rate

**FIGURE 1:**
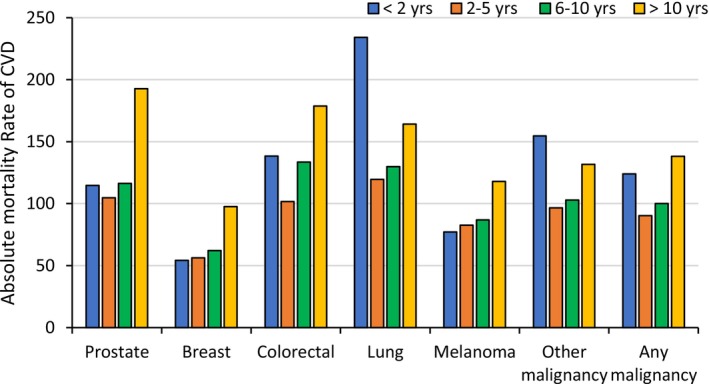
Cardiovascular disease (CVD) absolute mortality rate (per 10,000 person‐years) among people with cancer in New South Wales between 1985 and 2020, stratified by time since cancer diagnosis.

The CVD AMR per 10,000 person‐years was higher in males (136) than in females (100, Table 2). The highest CVD AMR was observed for people with lung cancer (182), those diagnosed with distant metastases (158), those aged 80 years or over (196) and 10 years after cancer diagnosis (152). The observed all‐cause AMR was 828 (per 10,000 person‐years) over the period 1985–2020, including 589 for cancer and 119 for CVD. In terms of time since cancer diagnosis, the highest CVD AMR was observed at > 10 years since cancer diagnosis, followed by within < 2 years of cancer diagnosis, except for people with breast cancer and melanoma, who had moderately increased CVD AMR up until 10 years and a sharp increase after 10 years (Figure 1). The CVD AMRs within increasing time periods after their cancer diagnosis were: 124 (per 10,000 person‐years) at < 2 years, 90 at 2–5 years, 103 at 6–10 years, and 152 at more than 10 years.

**TABLE 2:** Distribution of cancer cases, absolute mortality rate per 10,000 person‐years (1985–2019) and standardised mortality ratio by patients' characteristics in New South Wales.

Characteristics	Cancer cases	Absolute mortality rate			Standardised mortality ratio^a^	
		All‐cause	Cancer	CVD	CVD	All‐cause
Sex						
Female	390,986	712	511	100	0.8 (0.8, 0.81)	2.51 (2.5, 2.52)
Male	482,358	937	662	136	0.95 (0.94, 0.96)	2.33 (2.32, 2.34)
Age group						
40–59	248,783	680	573	39	1.13 (1.07, 1.19)	13.33 (13.23, 13.44)
60–79	488,856	829	580	123	0.98 (0.97, 0.99)	3.6 (3.59, 3.62)
> 80	135,705	995	632	196	0.84 (0.83, 0.85)	1.45 (1.44, 1.46)
Year after diagnosis						
0–2	284,878	1856	1611	124	1.17 (1.15, 1.19)	7.3 (7.28, 7.33)
2–5	166,610	636	464	90	0.84 (0.83, 0.86)	2.39 (2.37, 2.4)
6–10	168,242	451	249	103	0.83 (0.81, 0.84)	1.41 (1.4, 1.42)
> 10	253,614	518	191	152	0.81 (0.8, 0.82)	1.02 (1.02, 1.03)
Cancer stage						
Distant	130,710	4805	4468	158	1.31 (1.27, 1.35)	15.71 (15.62, 15.8)
Regional	178,359	832	625	102	0.86 (0.84, 0.87)	2.76 (2.74, 2.78)
Localised	354,767	466	255	106	0.82 (0.81, 0.83)	1.39 (1.39, 1.4)
Unknown	209,508	988	671	157	0.97 (0.96, 0.98)	2.39 (2.37, 2.4)
Cancer type						
Prostate	144,805	542	284	135	0.85 (0.84, 0.86)	1.21 (1.2, 1.22)
Breast	119,024	366	219	74	0.73 (0.72, 0.75)	1.54 (1.53, 1.56)
Colorectal	116,265	850	565	148	0.84 (0.83, 0.85)	1.94 (1.93, 1.96)
Lung	83,245	3949	3548	182	1.42 (1.37, 1.47)	11.79 (11.71, 11.88)
Melanoma	83,191	372	181	99	0.79 (0.78, 0.81)	1.16 (1.15, 1.17)
Other	326,814	1207	935	126	1.02 (1.01, 1.04)	3.87 (3.86, 3.89)
Any malignancy	873,344	828	589	119	0.88 (0.87, 0.89)	2.4 (2.39, 2.41)

^a^ Compared to the New South Wales general population.

**FIGURE 2:**
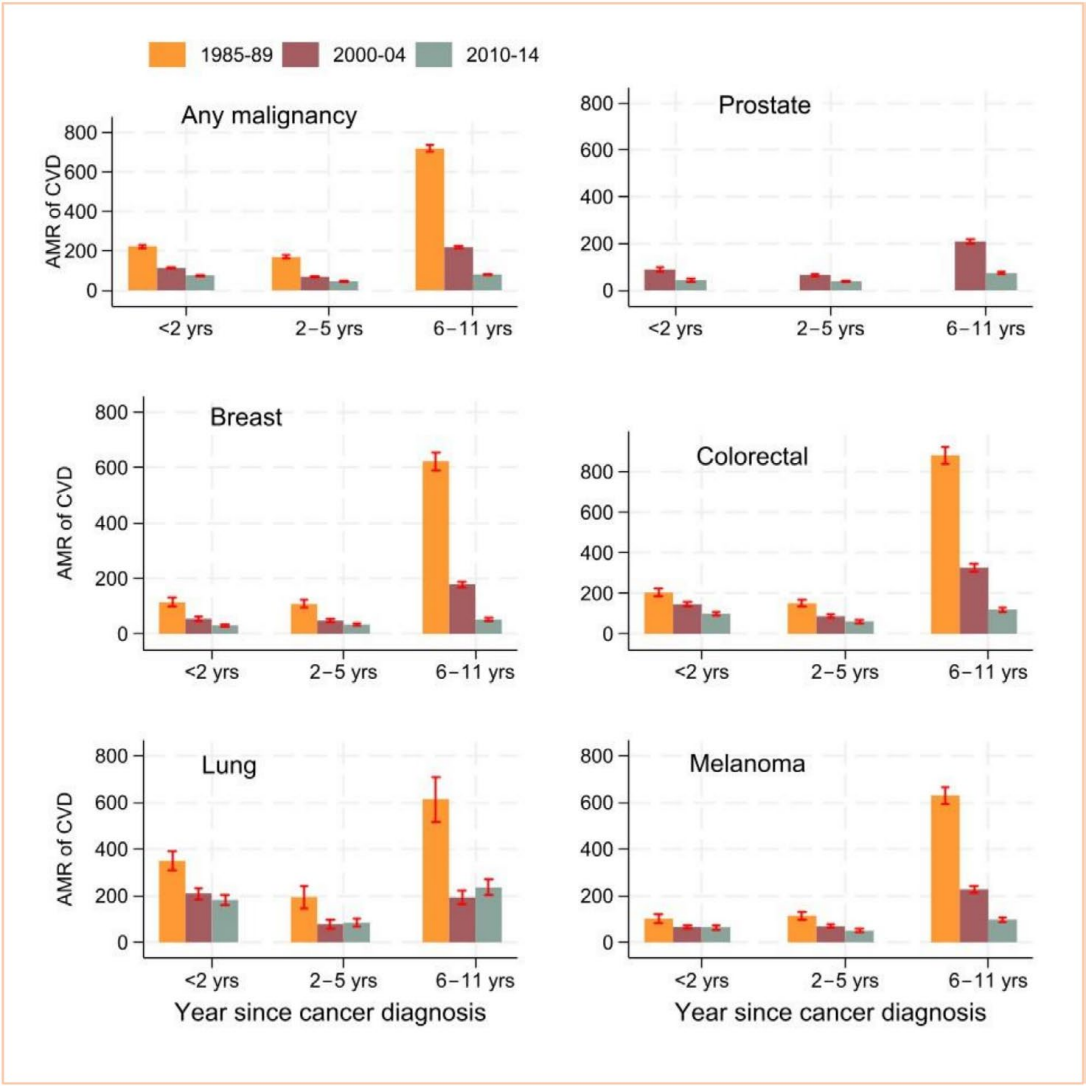
Trend in absolute mortality rate (AMR, per 10,000 person‐years) of cardiovascular disease (CVD) in three sub‐cohorts who were diagnosed with cancer in different periods (1985–1989, 2000–2004 and 2010–2014) stratified by time since cancer diagnosis.

In terms of temporal trends over calendar times, CVD AMRs by time after cancer diagnosis declined over the study period, with variations by cancer types (Figure 2). For example, for those who died within 2–5 years after their cancer diagnosis, the CVD AMR was 224 (per 10,000 person‐years) for those diagnosed in 1985–1989, and 76 for those diagnosed in 2010–2014. Cancer AMRs also declined over the study period: the cancer AMR was 1237 for those diagnosed in 1985–1989, and 562 for those diagnosed in 2010–2014 (Table S3).

### 3.2 Standardised Mortality Ratio

While the all‐cause mortality (SMR = 2.40, 95% CI: 2.39, 2.41) was very high, CVD mortality (SMR = 0.88, 95% CI = 0.87, 0.89) was significantly lower among people with cancer diagnosis compared to the NSW general population (Table 2). People with lung cancer had the highest all‐cause (SMR = 11.79, 95% CI: 11.71, 11.88) and CVD mortality (SMR = 1.42, 95% CI = 1.37, 1.47). Elevated SMRs of CVD were also observed for the period ≤ 2 years after cancer diagnosis (SMR = 1.17, 95% CI: 1.15, 1.19) and for those diagnosed with distant metastases (SMR = 1.31, 95% CI: 1.27, 1.35).

Table 3 presents the SMR (95% CI) for all‐cause and CVD across three different sub‐cohorts who were diagnosed with cancer in three different calendar periods and were followed up for 6–11 years after their cancer diagnosis. The results reveal that both all‐cause (SMR = 4.59, 95% CI: 4.55, 4.63) and CVD (SMR = 1.13, 95% CI: 1.07, 1.20) mortality were significantly higher for those diagnosed with cancer in 1985–1989 compared to the general population, with variation by cancer types. The corresponding figures for those diagnosed with cancer in 2010–2014 were 2.94 (95% CI: 2.92, 2.96) and 0.67 (95% CI: 0.65, 0.73), respectively. We also observed that the CVD SMR varied by cancer type and time since cancer diagnosis.

**TABLE 3:** Cardiovascular disease standardised mortality ratio (CVD SMR) and all‐cause standardised mortality ratio (all‐cause SMR) across three sub‐cohorts, and the whole cohort compared to the general population in New South Wales, Australia.

Characteristics	SMR		SMR		SMR	
	1985–1989 (*n* = 74,880)		2000–2004 (*n* = 124,108)		2010–2014 (*n* = 157,045)	
	CVD	All‐cause	CVD	All‐cause	CVD	All‐cause
Sex						
Female	0.88 (0.85, 0.92)	4.79 (4.72, 4.86)	0.91 (0.88, 0.94)	4 (3.96, 4.05)	0.77 (0.74, 0.81)	3.32 (3.28, 3.36)
Male	1.10 (1.06, 1.13)	4.46 (4.41, 4.51)	1.06 (1.03, 1.09)	3.27 (3.24, 3.3)	0.91 (0.88, 0.94)	2.72 (2.69, 2.74)
Age group						
40–59	1.08 (0.93, 1.22)	22.44 (22, 22.88)	1.26 (1.09, 1.43)	19.52 (19.2, 19.88)	1.12 (0.96, 1.28)	15.66 (15.4, 15.96)
60–79	1.05 (1.02, 1.09)	5.62 (5.56, 5.68)	1.03 (1.00, 1.07)	4.95 (4.90, 5.00)	0.93 (0.89, 0.97)	4.42 (4.37, 4.46)
80 and over	0.94 (0.90, 0.97)	1.72 (1.68, 1.76)	0.97 (0.94, 0.99)	1.67 (1.65, 1.70)	0.81 (0.79, 0.84)	1.49 (1.47, 1.51)
Year since diagnosis						
< 2	1.20 (1.16, 1.25)	7.29 (7.21, 7.37)	1.37 (1.33, 1.42)	7.90 (7.82, 7.97)	1.04 (1.00, 1.08)	6.82 (6.76, 6.89)
2–5	0.93 (0.89, 0.97)	2.95 (2.89, 3.01)	0.92 (0.88, 0.95)	2.39 (2.35, 2.43)	0.61 (0.58, 0.64)	1.90 (1.87, 1.93)
6–11	0.86 (0.82, 0.90)	1.69 (1.64, 1.73)	0.67 (0.64, 0.70)	1.38 (1.36, 1.41)	0.94 (0.91, 0.98)	1.38 (1.36, 1.40)
Stage at diagnosis						
Distant	1.51 (1.37, 1.65)	23.01 (22.6, 23.4)	1.87 (1.71, 2.02)	24.41 (24.0, 24.77)	1.36 (1.24, 1.48)	19.74 (19.47, 20)
Regional	0.93 (0.87, 0.99)	5.84 (5.73, 5.95)	0.93 (0.87, 0.98)	4.03 (3.96, 4.09)	0.84 (0.79, 0.88)	3.04 (2.99, 3.09)
Localised	0.94 (0.91, 0.98)	2.50 (2.46, 2.54)	0.88 (0.85, 0.91)	1.89 (1.86, 1.92)	0.77 (0.74, 0.80)	1.47 (1.45, 1.49)
Unknown	1.06 (1.01, 1.12)	4.81 (4.73, 4.90)	1.11 (1.07, 1.15)	3.27 (3.22, 3.32)	0.93 (0.88, 0.97)	2.52 (2.48, 2.56)
Cancer type						
Prostate^a^	1.13 (1.07, 1.20)	2.38 (2.31, 2.44)	0.87 (0.82, 0.91)	1.37 (1.34, 1.41)	0.69 (0.65, 0.73)	1.02 (1.00, 1.05)
Breast	0.78 (0.72, 0.84)	2.82 (2.74, 2.91)	0.77 (0.72, 0.83)	1.95 (1.90, 2.01)	0.70 (0.65, 0.76)	1.73 (1.68, 1.78)
Bowel	0.81 (0.76, 0.86)	3.41 (3.33, 3.49)	0.91 (0.86, 0.96)	2.82 (2.76, 2.88)	0.84 (0.79, 0.89)	2.39 (2.34, 2.44)
Lung	1.68 (1.51, 1.85)	20.15 (19.7, 20.5)	1.83 (1.65, 2.00)	18.15 (17.81, 18.5)	1.44 (1.30, 1.58)	13.76 (13.51, 14.01)
Melanoma	0.78 (0.71, 0.85)	1.66 (1.59, 1.73)	0.83 (0.78, 0.89)	1.37 (1.34, 1.41)	0.83 (0.77, 0.88)	1.29 (1.25, 1.33)
Any malignancy	1.00 (0.97, 1.02)	4.59 (4.55, 4.63)	1.00 (0.97, 1.02)	3.31 (3.28, 3.33)	0.85 (0.83, 0.87)	2.94 (2.92, 2.96)
**Whole cohort (1985–2019, *n* = 873,344) by time since cancer diagnosis**						
	**< 2 years**	**2–5 years**	**6–10 years**	**> 10 years**		
Prostate^a^	0.95 (0.82, 1.08)	0.84 (0.73, 0.95)	0.78 (0.69, 0.88)	0.84 (0.75, 0.93)		
Breast	0.74 (0.58, 0.90)	0.73 (0.59, 0.87)	0.74 (0.62, 0.87)	0.74 (0.65, 0.82)		
Bowel	1.04 (0.88, 1.19)	0.71 (0.59, 0.83)	0.78 (0.67, 0.89)	0.81 (0.72, 0.89)		
Lung	2.15 (1.77, 2.53)	1.16 (0.77, 1.55)	1.01 (0.63, 1.37)	0.84 (0.56, 1.11)		
Melanoma	0.79 (0.62, 0.96)	0.82 (0.66, 0.97)	1.00 (0.85, 1.15)	0.80 (0.70, 0.89)		
Any malignancy	1.17 (1.10, 1.24)	0.84 (0.79, 0.90)	1.00 (0.95, 1.05)	0.81 (0.77, 0.85)		

*Note:* The sub‐cohorts were followed up 6–11 years after cancer diagnosis with those diagnosed in 1985–1989 followed until 1995, those 2000–2004 until 2010 and those in 2010–2014 until 2020.

^a^ The prostate cancer incidence data between 1985 and 1989 may not be accurate as the number of incidences dramatically increased in 1990 following the adoption of the Prostate‐specific antigen (PSA) screening in 1989.

## 4 Discussion

In this large‐scale population‐wide study over 35 years of data, we investigated the CVD mortality pattern among people with cancer compared to the NSW general population stratified by time since cancer diagnosis and calendar period. The results reveal that while higher than expected CVD mortality was observed in people with cancer compared to the New South Wales general population within the first few years after cancer diagnosis, especially in those diagnosed with lung cancer and distant metastases at the time of cancer diagnosis, the overall CVD mortality was significantly lower, with substantial variation by cancer type, stage at diagnosis, time since cancer diagnosis and the calendar period. The CVD mortality burden was particularly high among males, those aged 80 and over and at 10 or more years after cancer diagnosis, which might reflect the presence of age‐related ischaemic heart disease, shared risk factors or possible late effects of cancer treatment‐related cardiotoxicity [8, 14, 17]. However, the proportional and absolute mortality of CVD declined in the most recent calendar period compared to the earliest calendar period. This likely reflects secular CVD trends, with possible reductions in cancer treatment‐related cardiotoxicity from advances in cardioprotective treatments [12], strategies [26] and guidelines [27].

The elevated CVD SMRs within the first few years after cancer diagnosis, those with distant metastases at the time of cancer diagnosis, and lung or other non‐major cancers were also reported in several population‐based studies in the USA [14, 15, 28, 29], Italy [30], and Japan [19]. Unlike some previous studies [14, 19, 29], we found significantly lower overall CVD SMRs compared to the NSW general population for people with any malignancy or major cancers (including prostate, breast, colorectal and melanoma). However, this result is consistent with Italian [30], and Korean [31] studies that reported significantly lower overall SMRs than the general population. A previous Australian study [18] reported that the risk of CVD mortality was not significantly different between people with cancer and the general population in Tasmania between 2006 and 2015. The discrepancy between our findings and some previous studies may be due to several reasons, including the composition of the study population in terms of time since cancer diagnosis and age range. Other reasons could be differences in the study population or period, healthcare system or treatment patterns, data reporting patterns across settings and possibly study methodology. These make it challenging to compare our findings with previous studies conducted in countries with different healthcare systems—all of which warrant further study.

Although the proportion of deaths due to CVD was lower among people diagnosed with lung cancer, distant metastases, and first few years following a cancer diagnosis, consistent with other studies, we observed elevated SMRs for these groups [14, 15, 30]. This may partly be explained by the excess mortality due to cancer in the first few years after cancer diagnosis and fewer CVD deaths in the comparable general population, as reflected in noticeably very high all‐cause mortality and the absolute number of CVD deaths. Increased CVD mortality in the first few years after diagnosis may be due to cardiac side effects from aggressive treatment shortly after cancer diagnosis or severe psychological distress following diagnosis [32, 33, 34]. It is also likely that some patients have existing CVD, which was exacerbated following a cancer diagnosis.

The observed versus expected CVD mortality for different subgroups of patients, including cancer types, across studies may not be comparable due to the differences in their comparator populations. Additionally, CVD mortality ratio for different cancer types may vary due to differences in treatment intensity, age and cancer stage at diagnosis, survival rates and age at death, and shared risk factors with CVD. For some cancer types, for example, colorectal cancer, we found CVD mortality was roughly a U‐shaped distribution: greater in the first year, declined in the next five years and again increased over time, partly due to the late effects of cancer treatment [15, 17]. Cancer‐specific analysis with more prognostic factors is needed to explore the complex interaction of CVD and cancer on patient outcomes, including mortality risk.

Consistent with a previous study [35], the common underlying causes of CVD deaths were due to IHD, cerebrovascular disease and hypertensive or other heart disease across all major cancer types. IHD accounted for the largest proportion of CVD deaths, with the highest proportion among males with lung cancer (54%), followed by prostate cancer (49%). This proportion is noticeably higher than the corresponding figure for the general population, which is 41% [36]. However, the cancer cohort is likely to be older, which may partly explain the differences. Unlike a recent US‐based study [35], we observed a moderately lower proportion of IHD deaths among those aged ≥ 80 at the time of cancer diagnosis across major cancer types but an increased proportion of other CVD deaths as well as other non‐cancer deaths. This may partly be explained by increased cancer and other causes of death among them. Our finding is consistent with the CVD death patterns in the Australian general population, published by the Australian Bureau of Statistics [37]. The variation with the US‐based study may be explained by the study population or reporting differences between Australia and the US.

The key strength of our study is that we report over 35 years of CVD mortality data among 873,344 people with cancer and assess their risk compared to the NSW general population, using the population‐wide NSW Cancer Registry linked with the cause of death data spanning from 1985 to 2020. Our study has some limitations. First, for the CVD mortality reported in this study we only considered a single underlying cause of death; among people with cancer, cancers are typically reported as the underlying cause of death and occur less frequently among associated causes of death [38]. Analyses incorporating multiple causes of death are likely to provide a more comprehensive picture of the role of CVD in mortality among people with cancer. Second, our estimates of observed versus expected CVD deaths did not allow for competing causes of death. Our study population is at much higher risk of dying from cancer than the general population. This would also mean that the estimated SMRs for CVD mortality are not reflective of the underlying CVD burden in people diagnosed with cancer. Third, our analysis aimed to quantify mortality patterns rather than establish causality. The SMRs were based only on age‐, sex‐ and calendar year‐specific mortality rates; further statistical adjustments were not done. Finally, caution is needed when comparing SMRs across subgroups, as subgroups may not be homogeneous in age distribution.

## 5 Conclusion

This large‐scale population‐wide study demonstrates that higher than expected CVD mortality was observed in people with cancer compared to the NSW general population within the first few years after cancer diagnosis, especially in those diagnosed with lung cancer and distant metastases. The analysis of the time trend shows that the observed absolute relative CVD mortality significantly declined in the recent period (2010–2020) following cancer diagnosis in 2010–2014. Although causality cannot be certain, given the possibility of shared risk factors for CVD and cancer impacting our findings, these results highlight the importance of early detection, improvement in cancer treatment‐related cardiotoxicity, and the treatment and care of CVD in people with a cancer diagnosis. Further cancer‐specific comprehensive studies, considering the effects of shared risk factors, history of CVD, coexisting conditions, and cancer treatment‐related cardiotoxicity, may beneficially inform guidelines for the prevention and care of CVD among people with cancer, which could lead to a reduction in mortality.

## Author Contributions

**Md Mijanur Rahman:** conceptualization, investigation, methodology, software, data curation, formal analysis, project administration, resources, visualisation, writing – review and editing, writing – original draft, validation. **Karen Canfell:** conceptualization, investigation, funding acquisition, methodology, writing – review and editing, supervision, resources. **Katy Bell:** conceptualization, investigation, writing – review and editing, methodology, validation, supervision. **Grace Joshy:** conceptualization, investigation, writing – review and editing, methodology, validation, supervision. **Michael David:** conceptualization, investigation, writing – review and editing, methodology, resources, supervision. **Anne Cust:** conceptualization, investigation, funding acquisition, writing – review and editing, methodology, resources. **David Goldsbury:** conceptualization, investigation, writing – review and editing, methodology, validation, visualisation, project administration, resources, data curation. **Bogda Koczwara:** conceptualization, investigation, writing – review and editing. **Emily Banks:** conceptualization, investigation, writing – review and editing, methodology, supervision. **Xue Qin Yu:** conceptualization, investigation, funding acquisition, writing – review and editing, methodology, resources, supervision, project administration.

## Disclosure

A.C., K.B., K.C. and E.B. are supported by National Health and Research Council of Australia Investigator Grants (NHMRC; GNT2008454, GNT1174523, APP1194679 and APP2017742, respectively). K.C. is co‐PI of an investigator‐initiated trial of cervical screening, ‘Compass’, run by the ACPCC, a government‐funded not‐for‐profit charity. Compass receives infrastructure support from the Australian government and the ACPCC has received equipment and a funding contribution from Roche Molecular Diagnostics, USA. Karen Canfell is also co‐PI on a major implementation programme Elimination of Cervical Cancer in the Western Pacific which has received support from the Minderoo Foundation and equipment donations from Cepheid Inc.

## Conflicts of Interest

The authors declare no conflicts of interest.

## Supporting information


**Table S1:** International Classification of Diseases (ICD) 9th revision and 10th revision codes for cancer and cardiovascular diseases (CVD) as the underlying causes of death.
**Table S2:** Type of cardiovascular disease (CVD) mortality among people with cancer by sex, age and calendar year between 1985 and 2020 in New South Wales, Australia.
**Table S3:** Absolute mortality rate (per 10,000 person‐years) by the underlying cause of death in three sub‐cohort 1985–1989, 2000–2004 and 2010–2014 with follow‐up until 1995, 2010 and 2020, respectively.

## Data Availability

This data analysis was conducted under conditions approved by the relevant ethics committee(s). As a condition of approval, data are not shareable. Access to data by other individuals or agencies would require appropriate ethical approvals to be in place.
