# Supplementary material for: Cardiovascular Disease Mortality Patterns Among People With Cancer in New South Wales, Australia: A Population‐Wide Data Linkage Study
Source: Cancer Med. 2026 Mar 6;15(3):e71568. doi: 10.1002/cam4.71568 (PMC12965825; doi:10.1002/cam4.71568)
Supplement: Supplementary file 1 — Table S1: International Classification of Diseases (ICD) 9th revision and 10th revision codes for cancer and cardiovascular diseases (CVD) as the underlying causes of death. Table S2: Type of cardiovascular disease (CVD) mortality among people with cancer by sex, age and calendar year between 1985 and 2020 in New South Wales, Australia. Table S3: Absolute mortality rate (per 10,000 person‐years) by the underlying cause of death in three sub‐cohort 1985–1989, 2000–2004 and 2010–2014 with follow‐up until 1995, 2010 and 2020, respectively. [file CAM4-15-e71568-s001.docx]

**Supplementary Table 1:** International Classification of Diseases (ICD) 9th revision and 10th revision codes for cancer and cardiovascular diseases (CVD) as the underlying causes of death

| **CVD type** | **ICD-9 codes (1985-1996)** | **ICD-10 codes (1997-2020)** |
| --- | --- | --- |
| Ischemic heart disease | 410-414 | I20-I25 |
| Pulmonary /other heart disease | 415-429 | I26-I50 |
| Hypertensive | 401-405 | I10-I13 |
| Cerebrovascular disease | 430-438 | I60-I69 |
| Other CVD | 390-400, 406-409, 439-459 | I00-I09, I14-I19, I51-I59, I70-I99 |

**Supplementary Table 2:** Type of cardiovascular disease (CVD) mortality among people with cancer by sex, age and calendar year between 1985 and 2020 in New South Wales, Australia

| Type of CVD by cancer type |  | Sex | |  | Age at death | | |
| --- | --- | --- | --- | --- | --- | --- | --- |
|  |  | Female | Male |  | 40-59 | 65-79 | ≥ 80 |
| **Any malignancy** | n | 30305 | 43428 |  | 1470 | 23435 | 48829 |
| Ischemic heart disease |  | 40% | 49% |  | 47% | 49% | 43% |
| Pulmonary/hypertensive/other heart disease |  | 12% | 10% |  | 15% | 11% | 11% |
| Cerebrovascular disease |  | 15% | 11% |  | 9% | 12% | 13% |
| Other |  | 32% | 30% |  | 27% | 28% | 33% |
| **Prostate** | n | _ | 16397 |  | 89 | 5073 | 11235 |
| Ischemic heart disease |  | _ | 49% |  | 61% | 52% | 47% |
| Pulmonary/hypertensive/other heart disease |  | _ | 10% |  | 11% | 10% | 9% |
| Cerebrovascular disease |  | _ | 11% |  | 5% | 10% | 12% |
| Other |  | _ | 30% |  | 22% | 28% | 32% |
| **Breast** | n | 9195 | _ |  | 150 | 2133 | 6912 |
| Ischemic heart disease |  | 39% | _ |  | 37% | 44% | 38% |
| Pulmonary/hypertensive/other heart disease |  | 12% | _ |  | 16% | 12% | 12% |
| Cerebrovascular disease |  | 15% | _ |  | 8% | 14% | 16% |
| Other |  | 33% | _ |  | 33% | 31% | 35% |
| **Colorectal** | n | 5966 | 6725 |  | 149 | 3471 | 9071 |
| Ischemic heart disease | % | 40% | 49% |  | 49% | 50% | 43% |
| Pulmonary/hypertensive/other heart disease |  | 12% | 11% |  | 17% | 12% | 11 |
| Cerebrovascular disease |  | 15% | 11% |  | 8% | 11% | 14% |
| Other |  | 33% | 30% |  | 21% | 28% | 33% |
| **Lung** | n | 1064 | 2339 |  | 146 | 1953 | 1304 |
| Ischemic heart disease | % | 43% | 54% |  | 39% | 53% | 49% |
| Pulmonary/hypertensive/other heart disease |  | 11% | 10% |  | 15% | 9% | 10% |
| Cerebrovascular disease |  | 16% | 11% |  | 13% | 12% | 13% |
| Other |  | 30% | 26% |  | 25% | 26% | 28% |
| **Melanoma** | n | 3455 | 5279 |  | 126 | 2213 | 6395 |
| Ischemic heart disease | % | 38% | 48% |  | 54% | 49% | 42% |
| Pulmonary/hypertensive/other heart disease |  | 12% | 10% |  | 12% | 11% | 11% |
| Cerebrovascular disease |  | 15% | 10% |  | 6% | 11% | 13% |
| Other |  | 35% | 31% |  | 29% | 29% | 34% |

**Supplementary Table 3:** Absolute mortality rate (per 10,000 person-years) by the underlying cause of death in three sub-cohort 1985-89, 2000-04 and 2010-14 with follow-up until 1995, 2010 and 2020, respectively.

| Characteristics | Cohort: 1985-89 | | |  | Cohort: 2000-04 | | |  | Cohort: 2010-14 | | |
| --- | --- | --- | --- | --- | --- | --- | --- | --- | --- | --- | --- |
|  | Cancer | CVD | Other |  | Cancer | CVD | Other |  | Cancer | CVD | Other |
| Sex |  |  |  |  |  |  |  |  |  |  |  |
| Female | 978 | 144 | 73 |  | 674 | 93 | 88 |  | 545 | 58 | 89 |
| Male | 1518 | 235 | 144 |  | 821 | 133 | 128 |  | 576 | 75 | 111 |
| Age group |  |  |  |  |  |  |  |  |  |  |  |
| 40-59 | 965 | 39 | 47 |  | 557 | 18 | 36 |  | 414 | 13 | 38 |
| 60-79 | 1342 | 208 | 118 |  | 762 | 101 | 103 |  | 530 | 50 | 85 |
| 80 and over | 1393 | 447 | 200 |  | 1035 | 319 | 248 |  | 905 | 213 | 253 |
| Year since diagnosis |  |  |  |  |  |  |  |  |  |  |  |
| <2 | 2640 | 224 | 136 |  | 1740 | 152 | 142 |  | 1300 | 76 | 115 |
| 2 to 5 | 710 | 169 | 88 |  | 444 | 99 | 89 |  | 336 | 46 | 65 |
| 6 to 11 | 333 | 161 | 88 |  | 229 | 90 | 93 |  | 190 | 81 | 121 |
| Stage at diagnosis^€^ |  |  |  |  |  |  |  |  |  |  |  |
| Distant | 7851 | 324 | 209 |  | 5449 | 197 | 201 |  | 4366 | 110 | 191 |
| Regional | 1472 | 158 | 84 |  | 789 | 95 | 92 |  | 541 | 62 | 94 |
| Localised | 559 | 171 | 88 |  | 310 | 93 | 84 |  | 208 | 58 | 84 |
| Unknown | 1444 | 229 | 158 |  | 808 | 159 | 158 |  | 539 | 90 | 132 |
| Cancer type |  |  |  |  |  |  |  |  |  |  |  |
| Any malignancy | 1237 | 188 | 107 |  | 751 | 114 | 45 |  | 562 | 68 | 101 |
| Prostate^†^ | 988 | 383 | 193 |  | 287 | 120 | 102 |  | 156 | 54 | 70 |
| Breast | 472 | 112 | 47 |  | 228 | 61 | 46 |  | 186 | 40 | 55 |
| Bowel | 1050 | 181 | 91 |  | 715 | 135 | 120 |  | 590 | 95 | 121 |
| Lung | 6019 | 296 | 207 |  | 4644 | 215 | 242 |  | 3344 | 128 | 237 |
| Melanoma | 269 | 104 | 44 |  | 211 | 85 | 71 |  | 182 | 73 | 94 |
| ^†^ The prostate cancer incidence data between 1985-89 may not be accurate as the number of incidences dramatically increased in 1990 following the adoption of the Prostate-specific antigen (PSA) screening in 1989.  ^€^The spread of cancer (stage) at diagnosis was recorded in the NSWCR as localised, regional (adjacent organs or regional lymph nodes), distant metastases, and unknown stage. In this study, patients missing information on cancer stage at diagnosis were grouped with the unknown group. | | | | | | | | | | | |
